# Distributed Algorithm for Base Station Assignment in 4G/5G Machine-Type Communication Scenarios with Backhaul Limited Conditions

**DOI:** 10.3390/s20226553

**Published:** 2020-11-17

**Authors:** Edgar A. Esquivel-Mendiola, Hiram Galeana-Zapién, David H. Covarrubias, Edwin Aldana-Bobadilla

**Affiliations:** 1 Centro de Investigación y de Estudios Avanzados del I.P.N. (Cinvestav), Unidad Tamaulipas, Ciudad Victoria 87130, Mexico; hiram.galeana@cinvestav.mx; 2 Centro de Investigación Científica y de Educación Superior de Ensenada (Cicese), Ensenada 22860, Mexico; dacoro@cicese.mx; 3 Conacyt-Centro de Investigación y de Estudios Avanzados del I.P.N. (Cinvestav), Unidad Tamaulipas, Ciudad Victoria 87130, Mexico; edwyn.aldana@cinvestav.mx

**Keywords:** base station assignment, distributed algorithm, IoT, resource allocation

## Abstract

A progressive paradigm shift from centralized to distributed network architectures has been consolidated since the 4G communication standard, calling for novel decision-making mechanisms with distributed control to operate at the network edge. This situation implies that each base station (BS) must manage resources independently to meet the quality of service (QoS) of existing human-type communication devices (HTC), as well as the emerging machine type communication (MTC) devices from the internet of things (IoT). In this paper, we address the BS assignment problem, whose aim is to determine the most appropriate serving BS to each mobile device. This problem is formulated as an optimization problem for maximizing the system throughput and imposing constraints on the air interface and backhaul resources. The assignment problem is challenging to solve, so we present a simple yet valid reformulation of the original problem while using dual decomposition theory. Subsequently, we propose a distributed price-based BS assignment algorithm that performs at each BS the assignment process, where a novel pricing update scheme is presented. The simulation results show that our proposed solution outperforms traditional maximum signal to interference plus noise ratio (Max-SINR) and minimum path-loss (Min-PL) approaches in terms of system throughput.

## 1. Introduction

The internet of things (IoT) is considered to be the next information revolution, where everyday objects equipped with embedded sensors will process and transmit data from its environment to the Internet. The mining of the collected data in IoT is expected to support knowledge-based decisions in healthcare, smart cities, transportation, industrial automation, etc. In this context, cellular communication systems will play a key role in IoT use cases, where mobile nodes (sensing objects) require a wide coverage area, e.g., location and mobility tracking, fleet management, and mobile health IoT applications [1]. Towards supporting IoT devices, the cellular network standards are evolving in order to support the connectivity to resource-constrained machine type communication (MTC) devices from IoT. However, the efficient support of pervasive IoT applications in cellular networks faces different challenges, mainly because HTC traffic requirements have historically driven current network management mechanisms and radio access network (RAN) infrastructure. Because of the fundamentally different characteristics of MTC concerning the existing human type communications (HTC), it is crucial the design of novel resource management mechanisms to efficiently utilize network resources while maintaining a good quality of service (QoS) in IoT cellular scenarios [2]. Unlike HTC involving voice calls, messaging, and web browsing, MTC is characterized by an increased number of IoT devices, traffic direction, message size, transmission periodicity, and power constraints of IoT devices [3]. Taking this into account, we argue that resource management solutions must consider the following aspects as core requirements. First, the RAN evolution towards flat network architectures calls for novel resource allocation schemes to be distributed across base stations (BSs) at the network edge [4,5,6,7]. Second, it is of paramount importance the efficient support of MTC devices, while taking into account IoT application-specific QoS requirements, such as a minimum data rate. Third, the incorporation of massive MTC devices and enhanced broadband services in the existing cellular network infrastructure may lead to congestion situations in different segments of the RAN, including the backhaul and the mobile core network used to interconnect end devices to remote cloud data centers. Hence, backhaul-awareness must be considered in resource allocation processes in order to cope with a possible shortage of backhaul resources [8,9,10,11]. It has been proved that the backhaul network constitutes a new resource bottleneck as more spectral efficient air interface technologies have been developed and deployed in cellular networks [6]. Attending to these arguments, in this paper, we address the BS assignment problem whose aim is to determine the most appropriate BS to transmit/receive data to/from each mobile device. This problem’s importance lies in the fact that, if users are not correctly associated with BSs, then the resulting performance could be affected in terms of throughput, interference, energy consumption, and load imbalance in the radio interface. In this context, this paper aims to develop a distributed BS assignment algorithm that is suitable for multi-cell mobile wireless systems for the efficient support of machine-type communication scenarios and assuming limited backhaul conditions. In the following, we briefly analyze the relevant approaches proposed in the literature proposed to solve the BS assignment problem.

### 1.1. Related Works

**Figure 1:**
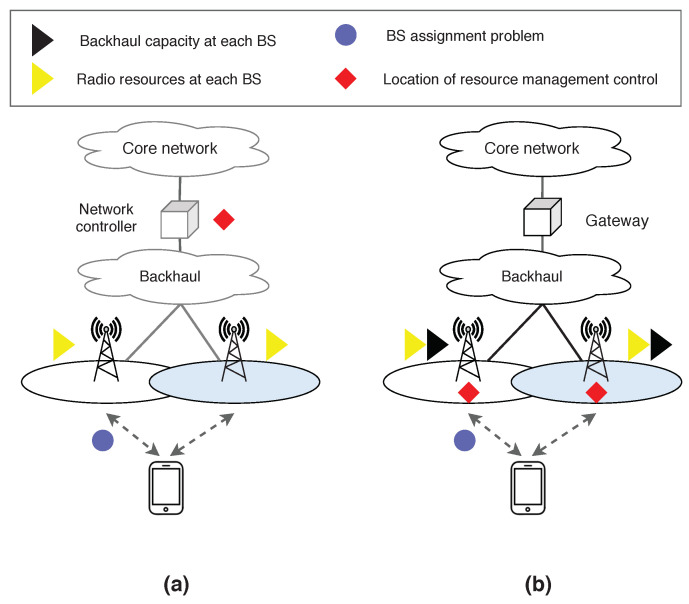
Generic access network architectures under (**a**) centralized and (**b**) distributed paradigms.

The BS assignment problem in mobile communication systems has been widely studied in the literature. Different approaches that range from greedy-based to heuristic-based algorithms have been proposed in the literature [12,13,14]. Two significant categories for the design and deployment of the BS assignment algorithms are identified in [15], namely the centralized and distributed approaches. On the one hand, in the centralized approach, as illustrated in Figure 1a, a single entity (e.g., radio network controller in 3GPP networks) is responsible for collecting the complete network state information and the resource demands from all devices in the system to solve the BS assignment problem. On the other hand, in the distributed approach that is illustrated in Figure 1b, each BS interacts with mobile devices and autonomously takes assignment decisions. Clearly, this latter approach implies lower implementation complexity and reduced signaling cost than the centralized solution and, hence, it is more suitable to large networks and ultra-dense network deployments [16].

In Table 1, we present a summary of the most relevant centralized and distributed BS assignment algorithms proposed in the literature so far. The related works are analyzed in terms of the modeling framework used to tackle the problem and the underlying mechanism that steers each related work’s assignment process. It is also highlighted the considered resource constraints at BSs, the device types (HTC, MTC), and the simulation setup from a system and device perspective. As surveyed in [15], the vast majority of existing BS assignment algorithms in the literature assume a centralized control. For instance, the works in [17,18,19,20,21,22,23] propose centralized heuristic algorithms in order to iteratively solve the BS assignment approach for the support of HTC devices in a single-cell network scenario with overlaid small-cells and radio/backhaul resource constraints. However, such solutions are not appropriate for distributed RAN deployments where decision making mechanisms, such as the BS assignment process, are located at the BS. We refer interested readers to [15] for a detailed analysis of centralized approaches. The rest of this section describes the distributed solutions, which is the main focus of this paper.

In [23], the joint optimization of the BS assignment and backhaul resource allocation is studied in a HetNet scenario. A heuristic algorithm is proposed, which iteratively performs the assignment process.

The work presented in [24] proposes a distributed pricing-based algorithm to solve the BS assignment problem, where a coordinate descent scheme is employed to update prices per resource unit. The authors in [25] also follow a pricing scheme, but the gradient descent method is used in order to update prices in the network. The authors in [26,27] study the BS assignment problem following pricing deployments, where the price per resource unit is updated according to interference and load conditions at BS. A common drawback of the previously described approaches is that they neglect potential backhaul constraints in the RAN, which implies that the user association is mainly driven by radio conditions and might result in unfeasible assignments from the backhaul viewpoint in scenarios where the backhaul capacity is the primary resource bottleneck (i.e., BSs with insufficient backhaul capacity to handle the aggregated data at the air interface). Besides, the described works do not consider machine type communication devices from the IoT ecosystem.

Because of the progressive evolution of IoT in cellular networks, recent works analyze the BS assignment problem in network scenarios, where both MTC and HTC devices are expected to be served. In [28], a congestion game approach is proposed in order to solve the BS assignment problem. Each user is modeled as a player who iteratively selects strategies that minimize its individual cost given the other players’ strategies. The authors in [29] propose a cognitive-inspired resource allocation approach. In this case, radio resources are assigned to HTC devices, whereas MTC devices opportunistically access to radio resources that are released by HTC devices. Similarly, the authors in [30] proposed a distributed Lagrange-based approach to tackle the BS assignment problem, when considering a HetNet cellular system. An approach based on game theory is studied in [31], where MTC devices are modeled with different QoS requirements.

Although the works described so far address the BS assignment problem over distributed architectures, they mainly focus on single macro-cell scenarios overlaid by small cells. The inter-cell interference conditions are not considered to be part of the BS assignment process. Furthermore, in terms of data rate requirements, homogeneous QoS requirements are commonly assumed in experimental evaluations considering both MTC and HTC devices. However, this assumption is not suitable for hybrid scenarios where IoT devices and cellular users are expected to be served by the cellular network Finally, the studied works generally assume ideal capacity conditions in the backhaul (unlimited capacity in backhaul links).

### 1.2. Contributions

This paper tackles the design and validation of a BS assignment algorithm that is suitable for cellular IoT networks. BSs are expected to independently determine users’ assignment solutions. We focus on cellular network scenarios, in which it is assumed that MTC and HTC devices co-exist in the same service area. The contributions of this paper are summarized, as follows:

- We propose a distributed pricing-based BS assignment approach based on a supply and demand scheme. The solution is independently executed in each BS following a bidding process that iteratively allocates resources to users willing to pay more for the available network resources.
- The core component of our proposal is a dynamic pricing update scheme that is based on a weighted utility function that involves users’ utilities and network load conditions.
- The proposed solution achieves good performance in terms of an available number of BS assignments, even in backhaul limited network scenarios, while also complying the data rate requirements of both MTC and HTC devices.

The remainder of this paper is organized as follows. In Section 2, we provide, what we consider, the most important elements to model and describe a downlink cellular system. In Section 3 we describe in detail the BS assignment problem and pose it as an optimization problem amenable to dual decomposition. In Section 4, we describe our algorithmic proposal for solving the BS assignment problem In Section 5, we present the experimental methodology and simulation results. Finally, in Section 6 we state some conclusions and discuss future work.

## 2. System Model

We consider a downlink cellular system with *N* BSs covering a geographical area in which *M* devices are needing to be served. Let *D* and *C* denote the set of MTC and HTC devices, respectively. The downlink data rate of each HTC device c∈C associated to BS *j* is denoted as $R_{cj}$. We also assume a minimum downlink data rate requirement that should be fulfilled (i.e., $R_{cj}\geq R_{c}^{min}$). Similarly, $R_{dj}$ denotes the downlink data rate of each MTC device d∈D, whereas $R_{dj}\geq R_{d}^{min}$ refers to each MTC device’s downlink minimum data requirement. Additionally, in line with long-term evolution (LTE)/4G and new radio (NR)/5G radio interfaces, we assume orthogonal frequency division multiple access (OFDMA) as an access method in the air interface so that the total system bandwidth *W* is divided into *K* resource blocks according to a frequency reuse pattern. Thus, a subset of $K_{j}$ resource blocks is assigned to each BS j∈N. Furthermore, each device i∈M is expected to be served by a single BS, which implies that fractional assignments are not allowed in our modeling. As for radio resources, each BS *j* has a maximum transmit power $P_{j}^{max}$. Without a loss of generality, we assume that the total transmission power of each BS is equally distributed, on average, over all available $K_{j}$ subchannels at BS *j*. This assumption is mainly intended to indicate that BSs are supposed to make use of all available subchannels in the same way (i.e., there is no subchannel more favored that another [32]). Hence, the mean interference power that is seen in each subchannel is expected to be, on average, the same. We also assume that each BS is constrained by a backhaul capacity of the wireless/wired link connecting each BS with the core network. Such backhaul link capacity at each BS could be defined, for instance, in terms of the maximum achievable data rate in the air interface. Over such a basis, the suitability of the assignment of the device *i* to BS *j* is modeled in terms of utility functions and resource cost functions, which have been successfully used in the modeling of resource allocation problems.

The above described system modelling is valid for any number of mobile devices and/or different minimum data rate requirements, as well as resource constraints. Hence, for a given system’s configuration, in this work the BS assignment problem can be regarded as finding the most appropriate assignment solution that satisfies each device’s QoS requirement and also fulfill resource constraints. In what follows, we elaborate on the considered utility and resource cost functions.

We first define a utility function $u_{ij}$ that represents the suitability of the assignment in terms of the signal-to-interference-plus-noise ratio (SINR) achieved by device *i* being assigned to BS *j*, over the resource block *k*, which is computed as:

(1) $$SINR_{i,j,k}=\frac{P_{i,j,k}G_{i,j,k}}{I_{i,j,k}+\eta }$$

where $P_{i,j,k}$ is the transmit power of BS *j* over the given resource block *k*, $G_{i,j,k}$ denotes the channel gain between device *i* and BS *j* over resource block *k*, which accounts for path loss, shadowing, and noise figure, η is the thermal noise level, and $I_{i,j,k}$ is the inter-cell interference experienced by the device at BS *j* on resource block *k*. Such inter-cell interference can be computed as:

(2) $$I_{i,j,k}=\sum_{n=1,n\neq j}^{n=N}P_{m\neq i,n,k}G_{i,n,k}$$

where $P_{m\neq i,n,k}$ is the transmission power of the *n* interfering BSs, on resource block *k* assigned to device m≠i. Accordingly, if device *i* is served by the BS *j*, the achievable transmission rate $r_{i,j,k}$ in terms of bps/Hz can be written as:

(3) $$r_{i,j,k}=\frac{W}{K_{j}}\log_{2}\left(1+SINR_{i,j,k}\right)$$

The utility function $u_{ij}$ quantifies the device’s satisfaction with respect to the achievable bit rate defined in Equation (3). Hence, the BS assignment choices having high SINR values lead to better utilities.

We also define a radio resource cost function, denoted to as $\alpha_{ij}=\frac{R_{i}^{\min}}{R_{ij}^{\max}}$, which relates the minimum data rate required by the device *i* (i.e, $R_{i}^{\min}$) and its maximum achievable rate at BS *j* computed as $R_{ij}^{\max}=\sum_{k=1}^{K_{j}}r_{i,j,k}$. Additionally, the backhaul resource cost $\beta_{ij}=\frac{R_{i}^{\min}}{C_{j}^{\mathrm{trans}}}$, denotes the relation between the minimum rate requirement of user *i* and the achievable backhaul capacity of BS *j*, resulting from the association of user *i* with BS *j*. Notice that $C_{j}^{\mathrm{trans}}$ denotes the available bandwidth of the wired/wireless backhaul link connecting to BS *j*.

## 3. Problem Formulation

Let $b_{ij}$ be a binary variable that denotes the absence or presence of an association between the *i*th device and *j*th BS and let $u_{ij}$ be the utility resulting from this association. The BS assignment problem can be posed as the search for those $b_{ij}$ that maximize the global network utility subject to several network constraints. This can be expressed as an optimization problem of the form:

(4a) $$\begin{array}{ccc} & \max_{b_{ij}} & \sum_{j=1}^{N}\sum_{i=1}^{M}u_{ij}b_{ij} \end{array}$$

(4b) $$\begin{array}{ccc} & s.t. & \sum_{i=1}^{M}\alpha_{ij}b_{ij}\leq 1,\;j=1,\ldots ,N\; \end{array}$$

(4c) $$\begin{array}{ccc} & & \sum_{i=1}^{M}\beta_{ij}b_{ij}\leq 1,\;j=1,\ldots ,N\; \end{array}$$

(4d) $$\begin{array}{ccc} & & \sum_{j=1}^{N}b_{ij}=1,\;i=1,\ldots ,M \end{array}$$

(4e) $$\begin{array}{ccc} & & b_{ij}\in \left\{0,1\right\} \end{array}$$

The constraints (4b) and (4c) ensure that the number of associated devices does not exceed the total radio and backhaul resources at BSs, respectively. The restriction (4d) means that each device can only be associated with one BS. Finally, recall that (4e) suggests that the association variable $b_{ij}$ must be binary. The problem (4a)–(4e) is a combinatorial optimization problem due to the binary variable $b_{ij}$, so that solving the problem with exact algorithms may be difficult, even for a small number of *N* and *M* [33,34]. Therefore, we reformulate the original BS assignment problem in order to make the problem more tractable. In particular, we aim to reduce the number of constraints and model the association process as a message passing based on pricing values of BSs. To this end, we make use of the dual decomposition theory that has been commonly used to design distributed algorithms in communication networks.

### 3.1. Dual Decomposition

The above problem can be expressed as a dual problem by coupling the resource constraints (4b) and (4c) with the objective function by introducing Lagrange multipliers, and keeping the constraints of the association:

(5) $$\begin{array}{c} \begin{array}{ccc} & \max_{b_{ij}} & \left\{\sum_{j=1}^{N}\sum_{i=1}^{M}u_{ij}b_{ij}-\sum_{j=1}^{N}\lambda_{j}\left(1-\sum_{i=1}^{M}\alpha_{ij}b_{ij}\right)-\sum_{j=1}^{N}\mu_{j}\left(1-\sum_{i=1}^{M}\beta_{ij}b_{ij}\right)\right\} \end{array} \end{array}$$

where $\lambda_{j}$ and $\mu_{j}$ are the positive Lagrange multipliers values associated with the radio and backhaul constraint on each BS, respectively. Subsequently, from problem (5), the Lagrange function can be obtained as:

(6) $$\begin{array}{c} \begin{array}{ccc} & L\left(b,\lambda ,\mu \right)=\max_{b_{ij}} & \left\{\sum_{j=1}^{N}\sum_{i=1}^{M}u_{ij}b_{ij}-\lambda_{j}\alpha_{ij}b_{ij}-\mu_{j}\beta_{ij}b_{ij}\right\} \end{array} \end{array}$$

The resulting dual problem for the primal problem is expressed as:

(7) $$\begin{array}{c} \min_{\lambda \geq 0,\mu \geq 0}g(\lambda ,\mu )=\left\{\begin{array}{cc} \max_{b} & L(b,\lambda ,\mu )\\ s.t. & \sum_{j=1}^{N}b_{ij}=1,\;i=1,\cdots ,M\\ & b_{ij}\in \left\{0,1\right\} \end{array}\right. \end{array}$$

In this sense, the maximization of the Lagrange function has the following analytical solution:

(8) $$\begin{array}{c} b_{ij}^{*}=\left\{\begin{array}{cc} 1, & \mathrm{if}\;\;j^{*}=\underset{j}{\mathrm{argmax}}\left(w_{ij}\right)\\ 0, & \mathrm{otherwise} \end{array}\right. \end{array}$$

where $b_{ij}^{*}$ denotes the assignment solution to user *i* and $w_{ij}$ is the weighted utility that device *i* can achieve if it is associated with BS *j*. It is worth noting that the obtained solution $b_{ij}^{*}$ obtained in Equation (8) meets the resource constraints at each BS *j* and the minimum data rate requirements of each device *i*, with $R_{ij}b_{ij}^{*}\geq R_{i}^{\min}$ where *i* could be either MTC or HTC device, corresponding to devices *d* and *c*, respectively. Notice that this latter aspect is achieved due to that the minimum rate requirement is involved in the computation of both the radio and backhaul resource costs.

The weighted utility is defined as the utility minus the price per resource unit on the radio and backhaul resources, which is formally expressed as:

(9) $$w_{ij}=u_{ij}-\lambda_{j}\alpha_{ij}-\mu_{j}\beta_{ij}$$

The weighted utility is used in order to steer the distributed BS assignment process, where the main challenge is to efficiently compute the pricing values $\lambda_{j}$ and $\mu_{j}$, denoting the Lagrange multipliers associated to radio and backhaul constraints, respectively. Details of the developed pricing update scheme are provided herein.

### 3.2. Pricing Update Scheme

In order to find the BS assignment solution given in Equation (8), a procedure to compute the appropriate pricing values $\lambda_{j}$ and $\mu_{j}$ is needed. In this regard, existing pricing-based schemes (see Table 1) reported in the literature rely on the definition of a step size value to incrementally adjust pricing values until a convergence criterion, in terms of load conditions and QoS is reached (as described later on). The step size is commonly defined as input to the existing distributed BS assignment algorithms, and its definition requires a fine tuning procedure during the design of the algorithm process. Unlike similar pricing-based approaches, the pricing values $\lambda_{j}$ and $\mu_{j}$ are independently updated at each BS *j* by means of a step size whose value depends on the observed load conditions. That is, there is no need for a fine tuning procedure to define the step size. Notice that the appropriate definition of such a step size value directly influences the number of iterations that are required to find the pricing values. In this regard, the main advantage of our approach is that pricing updates influence the BS assignment choice for each device based on two core aspects: (a) the maximum price users are willing to pay for the available network resources and (b) the observed weighted utility defined in Equation (8).

The pricing update procedure works, as follows. If a BS *j* exceeds its radio or backhaul resources, then the corresponding resource prices are updated as a means to steer the resource costly device $i^{{}^{\prime }}$ to a less loaded BS $j^{{}^{\prime }}$. Such a reassignment of the device could be achieved if price values at the serving BS *j* are updated to meet the condition $w_{i^{{}^{\prime }}j^{{}^{\prime }}}>w_{i^{{}^{\prime }}j}$. In short, the pricing update at BS *j* makes device $i^{{}^{\prime }}$ change its assignment preference to BS $j^{{}^{\prime }}$, where the highest weighted utility is obtained. It is worth noting that the pricing update should be performed in order to guarantee that only one device is reassigned to other BS. To this end, the price per radio resource unit at BS *j* should be updated, as follows:

(10) $$\lambda_{j}=\lambda_{j}+\Delta \lambda_{i^{{}^{\prime }}j}$$

where $\Delta \lambda_{i^{{}^{\prime }}j}$ is the minimum increase in the radio resource price to force device $i^{{}^{\prime }}$ to change its association request. Such a step size is derived using the weighted utility definition from Equation (9), and replacing Equation (10) into $w_{i^{{}^{\prime }}j^{{}^{\prime }}}>w_{i^{{}^{\prime }}j}$. Subsequently, the minimum increase of the radio resource price is expressed as $\Delta \lambda_{i^{{}^{\prime }}j}=\frac{u_{i^{{}^{\prime }}j}-\mu_{j}\beta_{i^{{}^{\prime }}j}}{\alpha_{i^{{}^{\prime }}j}}$. A similar procedure is applied to update the backhaul multiplier, as expressed by:

(11) $$\mu_{j}=\mu_{j}+\Delta \mu_{i^{{}^{\prime }}j}$$

where $\Delta \mu_{i^{{}^{\prime }}j}=\frac{u_{i^{{}^{\prime }}j}-\lambda_{j}\alpha_{i^{{}^{\prime }}j}}{\beta_{ij}}$ is defined as the minimum increase in backhaul resource price.

Note that the second term in Equations (10) and (11) refers to the step size used to update pricing values of radio and backhaul resources, respectively. We argue that such a minimum increase in pricing values allows us to efficiently explore the possible values that meet the resource assignment conditions.

## 4. Distributed BS Assignment Algorithm

The proposed BS assignment algorithm is based on the supply and demand scheme, where prices per resource unit are defined based on the information exchange between mobile devices and BSs. Subsequently, a bidding process is proposed in order to steer the BS assignment depending on the prices defined by each BS. The bidding process in our proposed solution is realized using two algorithms, one running on the mobile device and another one at the BS. Under this pricing framework, and the mapping of the original optimization problem into the dual problem, each BS *j* computes the prices defined in Equations (10) and (11). The underlying idea of our approach is that each device selects the serving BS based on the weighted utility. This, in turn, depends on the prices that are disseminated by BSs, which are updated as function of the demand experienced at each BS.

### 4.1. Overview of the BS Assignment Process

**Figure 2:**
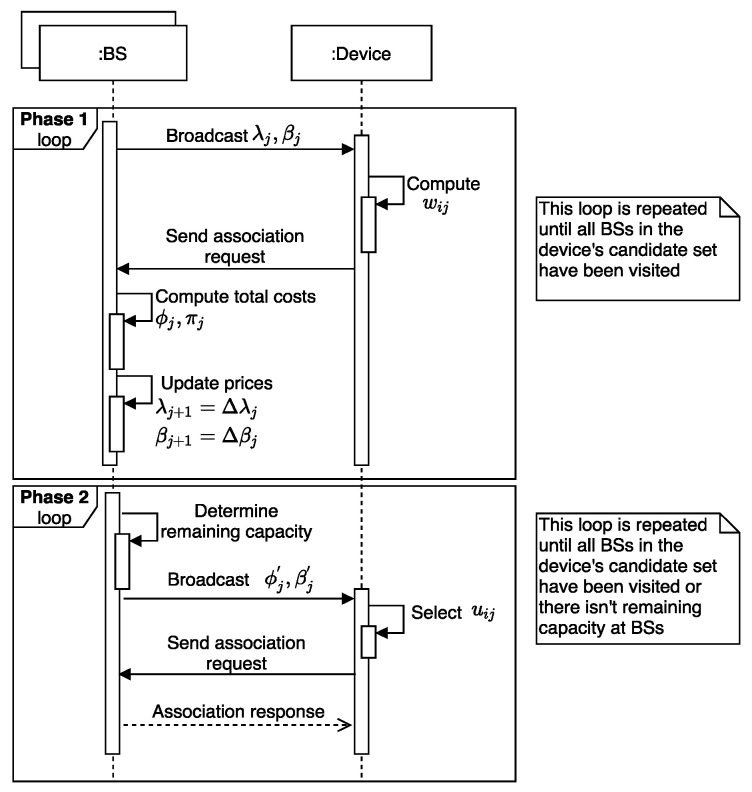
Sequence diagram of the BS assignment process performed based on the information exchange between each mobile device and its candidate BSs.

The overall BS assignment process is divided into two phases, as illustrated in Figure 2: (i) Phase 1 aims to find a feasible BS assignment solution following the proposed pricing scheme; and (ii) given the BS assignment solution from Phase 1, the aim of the Phase 2 is to exploit the remaining capacity at BSs to improve (if possible) the quality of the assignments in terms of network utility. A detailed description of the steps performed of the algorithm running at the BS and mobile device sides is given in Section 4.2 and Section 4.3, respectively.

### 4.2. Procedures at the BS

The pseudo-code of the algorithm running at each BS is illustrated in Algorithm 1. Firstly, each BS sets and disseminates the initial radio and backhaul prices. Subsequently, BS *j* gets association requests from devices and groups them in an association request set denoted to as $ar_{j}$. Each association request is assumed to include metrics related to the observed channel conditions at the device side. The total radio and backhaul costs are computed (step 6) to check if the current set of association requests does not exceed the resource constraints. In this step, if the radio or backhaul constraint is the most violated, the corresponding price is updated to alleviate the excess the load. In steps 8–12, the algorithm determines the device to be reassigned to another BS different that the requested one. To this end, the corresponding resource price (radio or backhaul) is increased (step 9–11) in such a way that only one device is moved. Notice that the device causing the highest cost is also the device with the minimum price increase according to step 11. Each BS independently chooses the device causing the minimum increase in the price and update the radio price according to Equation (10) (steps 14–16). In the end, if the stopping criterion is met BS stops updating the resource pricing, otherwise repeat step 3. The aforementioned process is repeated for updating the backhaul price when the associated constraint is exceeded (steps 18–27).

For the second phase, each BS computes its remaining capacity on the radio and backhaul resources. Thus, if there are available resources, then the BSs broadcast the remaining capacity on both radio and backhaul resources. Subsequently, BS receives association requests from devices, and sorts the requests $ar_{j}$ in terms of utility (steps 33–34). For each association request, BS determines whether device QoS requirements do not exceed the available capacity. More specifically, if device *i* is accepted by BS *j*, then the sum of its cost on radio $\alpha_{ij}$ and backhaul $\beta_{ij}$ should not exceed the total radio $\phi_{j}$ and backhaul $\pi_{j}$ costs. This phase is repeated until there are not available resources on BSs or devices have already sent an association request to all BSs on its candidate set.
**Algorithm 1:** BS’s algorithm
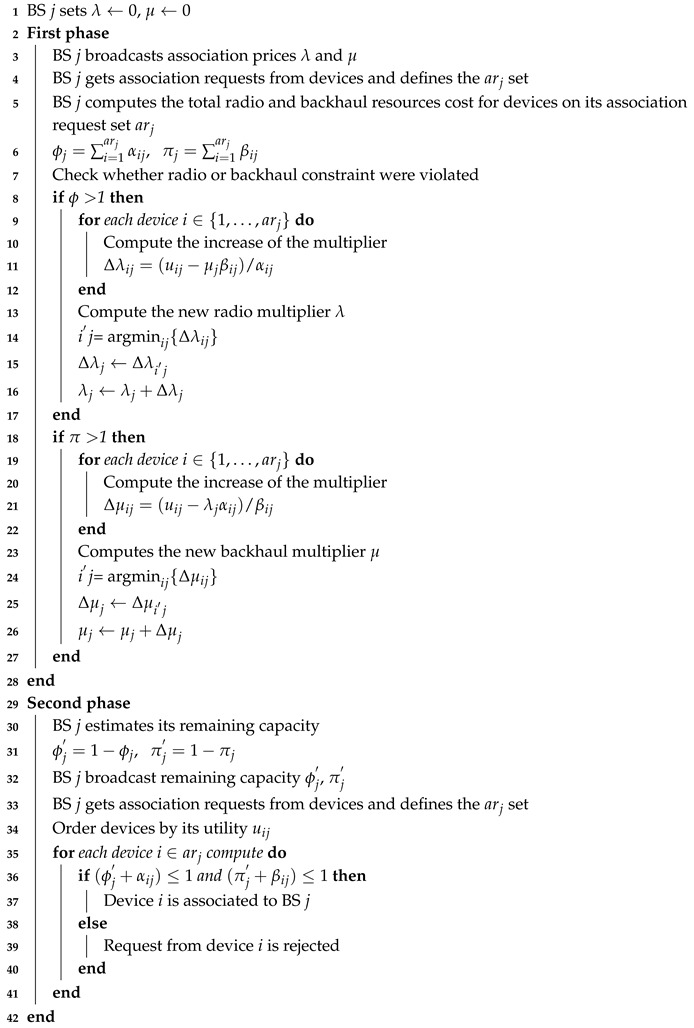


### 4.3. Procedures at the Mobile Device

As shown in Algorithm 2, each device initially computes a candidate set, denoted to as cs, with the set of BSs offering a path-loss within the margin between minimum offered path-loss and a path-loss margin (PLM). Next, once the device receives association prices from BSs, it computes for each BS on its cs the radio cost, utility, backhaul cost and weighted utility metrics. Subsequently, the device *i* selects the most appropriate BS $j^{*}$ according to the maximum weighted utility (step 8–9). The BS $j^{*}$ is the BS that offers the best relation of utility minus the association prices. After that, device *i* sends an association request to BS *i*, which includes the costs and utility metrics (step 10). The stopping criterion is reached when the device has already tried to be associated with each BS on its cs.
**Algorithm 2:** Device algorithm
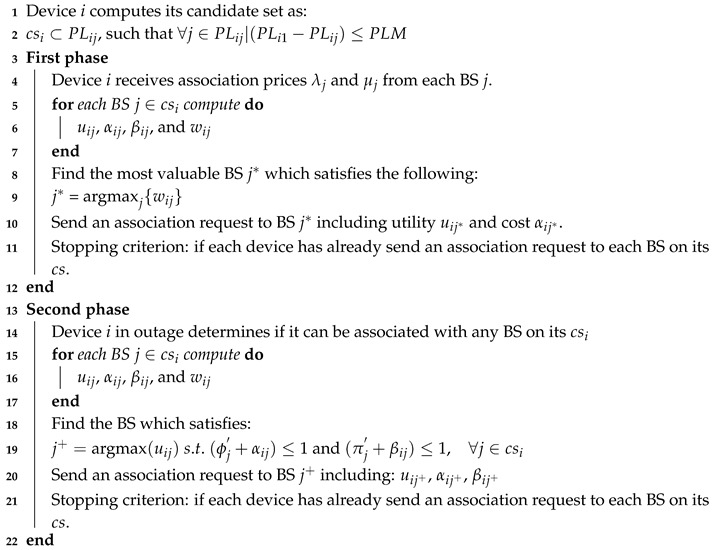


The second phase has been proposed to improve (if possible) the assignment solution found in phase 1. This is possible if there are available resources at BSs, and an improvement in the system utility is observed. Thus, each device receives current total radio and backhaul costs from all BSs, and determines whether it can improve its utility by being associated with another of the BSs on its cs. For that, the device checks whether the addition of its cost with the total cost of the BS does not exceed the maximum capacity on both radio and backhaul costs. Subsequenly, for each BS on its candidate set computes the utility, radio cost, and backhaul cost (step 15–17). The device determines the appropriated BS in which is observed an utility improvement and where the costs constraints are satisfied (step 18–19). Next, an association request is send to the BS $j^{+}$, which includes the metrics of utility and costs (step 20). The stopping criterion of this phase is reached when the device has tried to be associated with all pf the BSs on its candidate set.

## 5. Simulation Results

This section evaluates the performance of the proposed distributed price-based BS assignment algorithm. We consider a system that is composed of 19 hexagonal cells distributed in three tiers (one centric cell in first tier and two concentric tiers). The HTC devices and MTC devices are uniformly distributed over the coverage area. It is worth noting that the simulation results are presented in terms of the density of users per cell, where the total number of users in the system ranges from 190 to around 700 devices. Each device computes its candidate set when considering a PLM value of 6 dB, which implies that each BS whose path loss falls within such a range is considered as candidate BS. The BSs are configured with a maximum transmit power of 43 dBm. Furthermore, a bandwidth of 10 MHz is considered with a frequency reuse pattern of 3. The HTC devices and MTC devices have different downlink data rate requirements, which are defined as $R_{c}^{min}$ and $R_{d}^{min}$, respectively. The propagation losses are computed while using the Okumura–Hata model with the parameters given in Table 2. The log-normal shadowing is modeled with a standard deviation equal to 8 dB, and a noise figure of 5 dB is also considered. Based on the SINR, the modulation and coding scheme (MCS) and, consequently the achievable rate is taken from the look-up table provided in [8]. The cell’s radius has been computed while taking into account that the minimum SNR requirement of the MCS 1 is assured at the cell edge. The main simulation parameters are presented in Table 2. In our simulations, we evaluate the performance of the following algorithms: (a) Max-SINR, a greedy approach that assigns the devices with the BS that provides the highest SINR value [14]; (b) Min-PL, which assigns each device with the BS that provides the minimum path-loss [35], and (c) Price-based algorithm, which is the proposed approach in this paper.

Given a snapshot of the system with a distribution of devices over the coverage area, the algorithms are used to find a BS assignment solution. Subsequently, for each obtained solution, the following performance metrics are obtained:

- Feasibility. This experiment allows for us to measure the percentage of feasible solutions found by each algorithm. A solution is assumed to be feasible when the system’s constraints (i.e., radio and/or backhaul resources) are not exceeded.
- Throughput. Given a BS assignment solution, we compute the total system’s throughput as the sum of the data rate achieved by each device being served by a given BS.
- Resource costs. We compute each BS’s average sum cost to analyze if the algorithms violate the cost constraint.
- Rate degradation. This metric shows the data rate reduction experienced by devices when they are assigned to overloaded BS.

**Figure 3:**
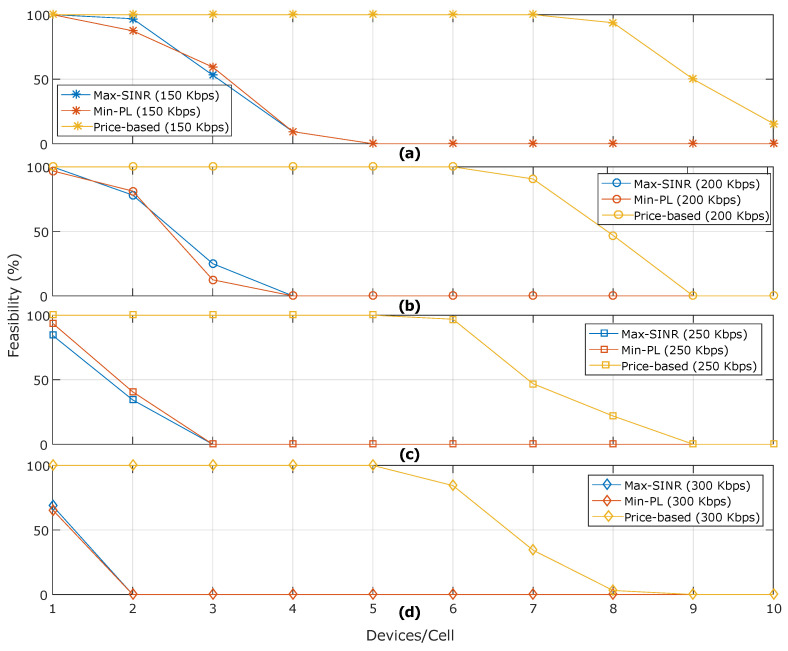
Feasible solutions (%) found by each algorithm under a backhaul capacity of 12 Mbps and different data rate requirements of human-type communication devices (HTC) devices: (**a**) 150 Kbps, (**b**) 200 Kbps, (**c**) 250 Kbps, and (**d**) 300 Kbps.

Figure 3 depicts the percentage of feasible solutions under the variation of the number of HTC devices per cell and a minimum rate requirement $R_{c}^{\min}=\left\{150,200,250,300\right\}$ Kbps, and assuming a minimum rate requirement of MTC devices of $R_{d}^{\min}=25$ Kbps. A solution is assumed to be feasible when the system’s resource constraints are not exceeded. It can be seen that, when the minimum rate requirement has increased, the percentage of feasible solutions is reduced for the three algorithms. For example, in the configuration of seven users per cell, our proposed algorithm achieves 100% for a rate requirement of 150 kbps, while a reduction of 60% is observed when the condition is of 300 kbps. Furthermore, the pricing-based solution has a better performance than greedy algorithms in terms of feasibility. For instance, taking Figure 3b as a basis, our proposed approach has a feasibility of 90% when there are seven devices per cell, while the Max-SINR and Min-PL algorithms achieve a 0% feasibility; this is because, in both algorithms, the resource capacity at BSs is exceeded.

**Figure 4:**
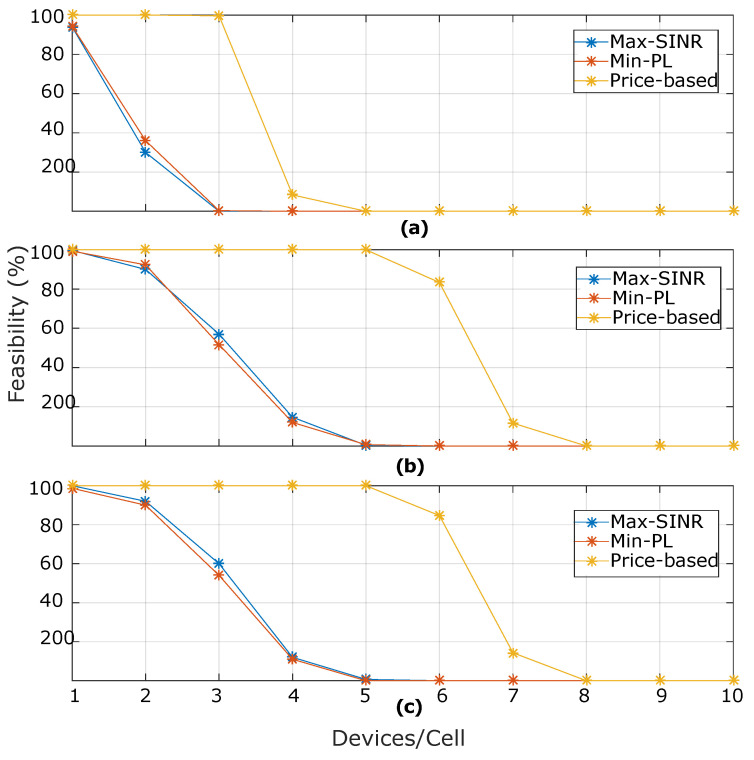
Feasible solutions (%) found by each algorithm under different backhaul capacity configurations (**a**) 4 Mbps, (**b**) 12 Mbps, and (**c**) 21 Mbps. The $R_{c}^{\min}$ requirement is assumed to be 250 Kbps for human-type communication devices (HTC) devices and $R_{d}^{\min}$= 25 Kbps for machine type communication (MTC) devices.

Figure 4 shows the percentage of feasible BS assignment solutions under the variation of the backhaul capacity of BSs. A solution is assumed to be feasible when all of the system’s resource constraints are satisfied. It is noted in the results that, for $R_{c}^{\min}$=250 Kbps, the percentage of feasible solutions is reduced for the three algorithms as the backhaul capacity is more limited. In the case of the six devices per cell and a backhaul capacity of 12 Mbps, our proposed approach has a percentage of 85%. In contrast, for a more restrictive scenario with a backhaul capacity of 4 Mbps, it has 0% feasibility. Even when there are available radio resources, the backhaul imposes a hard constraint on the assignment solution. From the results, even when the minimum required data rate is low, the reason for the excess in the backhaul resource constraints is that the assignment between a device *i* and a BS *j* implies a data rate transmission that is associated to the perceived SINR. More specifically, given the SINR value in the association device to BS, the assigned data rate is computed according to a modulation and coding method, which varies from 1.5 Mbps to 46.1 Mbps.

**Figure 5:**
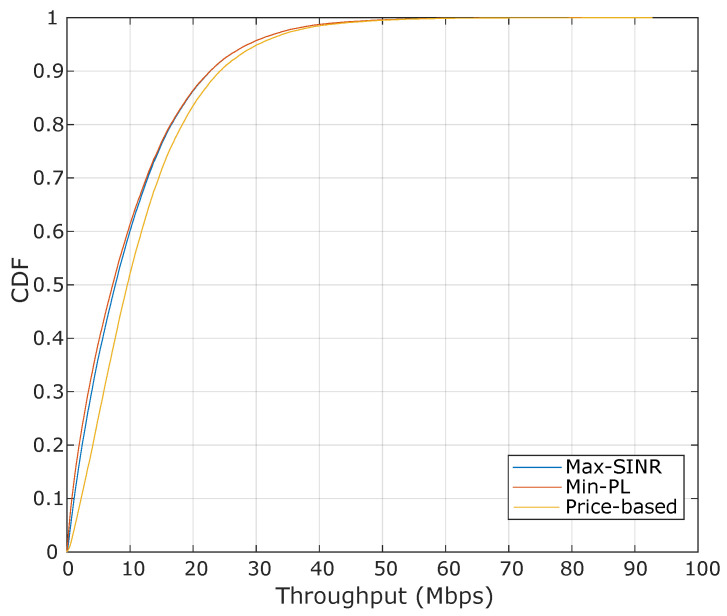
Cumulative distribution function (CDF) of the throughput achieved by each algorithm. The number of HTC and MTC devices per cell is set 10 and 20, respectively. The backhaul capacity is assumed with a value of 12 Mbps.

Figure 5 shows the cumulative distribution function (CDF) of the achieved throughput for the assignment solutions found by each algorithm. The number of HTC and MTC devices per cell is set to 10 and 20, respectively, which results in a total number of devices of 570 in the system. It is shown that the proposed pricing-based algorithm outperforms the two benchmark algorithms in terms of system’s throughput. For instance, for the pricing-based algorithm, 50% of devices achieved an efficiency of 9.5 Mbps, while, for the Max-SINR and Min-PL algorithms, the achieved throughput is about 7 Mbps. That is because the assignment found by Max-SINR and Min-PL algorithms exceed the BSs capacity, and the assigned devices experience a reduction in the achievable data rate.

**Figure 6:**
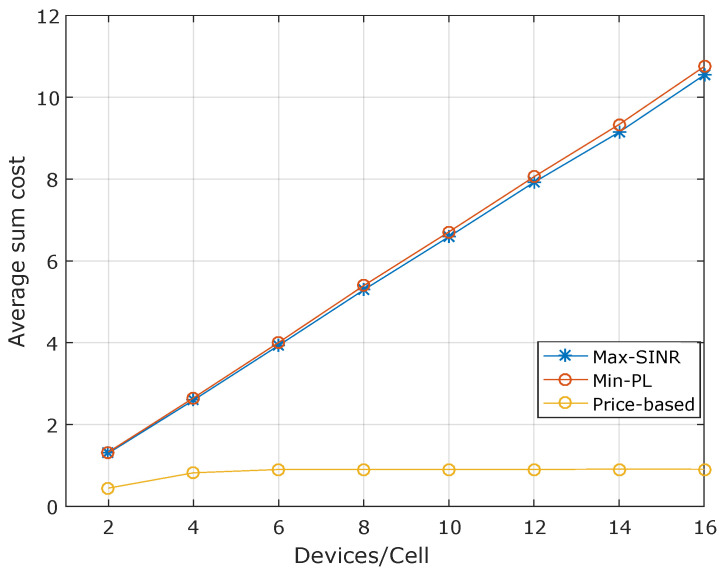
Average sum cost observed at the BSs as a result of the BS assignment solution found by each algorithm. The $R_{c}^{\min}$ requirement was set to 250 kbps for HTC devices and $R_{d}^{\min}$ of 25 Kbps for MTC devices.

**Figure 7:**
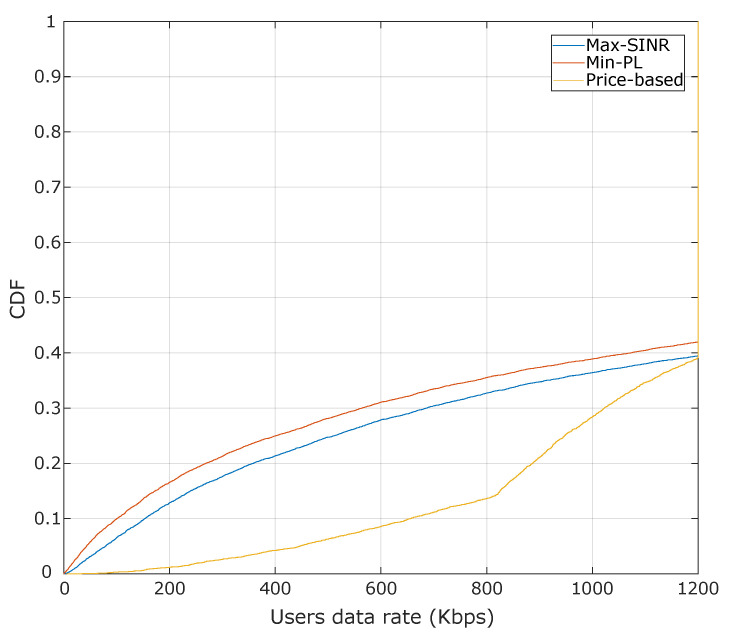
CDF of the data rate degradation for a $R_{c}^{\min}$ requirement of 600 Kbps for HTC devices and $R_{d}^{\min}$ of 30 Kbps for MTC devices. Also, the number of devices was set to 12 HTC devices and 25 MTC devices per cell. The system backhaul was considered to be limited with a backhaul capacity of 12 Mbps.

Furthermore, in terms of the average sum cost at BS, there is a significant difference between the greedy algorithms and our proposed approach. The price-based algorithm’s average sum cost is always below the maximum allowable cost of BSs, as illustrated in Figure 6. For instance, in eight devices, the average cost observed for the price-based algorithm is around to 1, while for the Max-SINR and the Min-PL algorithms, there is an average cost of 5.8. That is because the backhaul and radio capacity constraints steer the assignment process in the pricing-based approach. At the same time, the other algorithms do the assignment without considering such constraints. In this sense, the average sum costs of the greedy approaches reflect the number of resources that would be needed to support the provided BS assignment solution. We observe that such unfeasible BS assignment solutions lead to a user rate degradation situation, as presented in Figure 7. To be specific, we quantify data rate degradation experienced by users due to unfeasible BS assignment solutions (i.e., when the BS capacity is exceeded), which can be caused by the unbalance of the users’ distribution among the BS. In Figure 7, we observe that 84% of the users achieve a data rate of 800 Kbps for the pricing-based approach. In contrast, for the Max-SINR and the Min-PL algorithms, there is observed a degradation, since only 63% and 65% of the users achieve the data rate of 800 Kbps, respectively.

### Price-Based Algorithm vs. Centralized Approach

**Figure 8:**
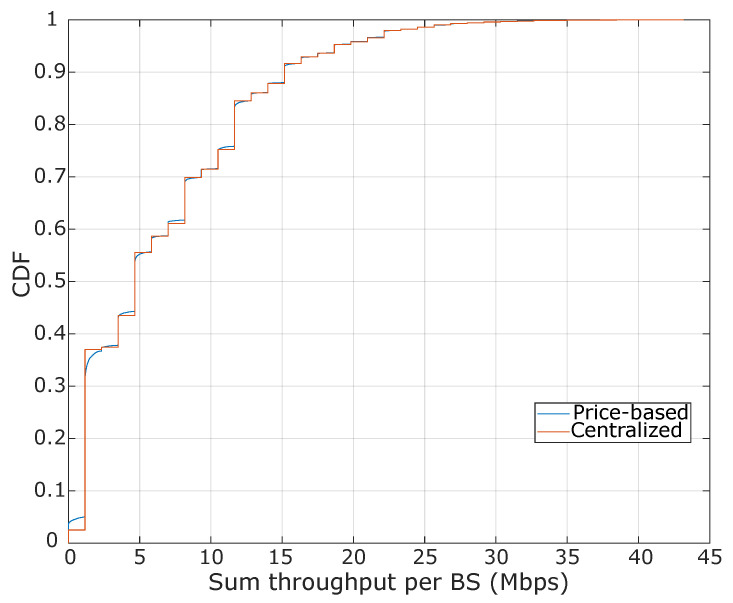
Average sum throughput per BS, where the system was configured with five devices (on average) uniformly distributed over each cell and with a $R_{c}^{\min}$ requirement of 250 Kbps.

Herein, we compare the proposed distributed algorithm with a centralized approach to solving the BS assignment problem. In particular, we analyze the performance achieved by each algorithm in terms of the sum throughput in the system, and also estimate the execution cost in terms of the total iterations that are required to provide a BS assignment solution. The obtained results are discussed in the sequel. We compared the pricing-based algorithm’s performance with the centralized BS assignment algorithm presented in. Both of the algorithms achieve similar behavior in terms of the average sum throughput, as observed in Figure 8. As mentioned before, our proposed solution only requires local information at each BS to perform the assignment process, contrary to the centralized approach in which the algorithm requires the complete network state information to decide the assignment and the resource distribution.

**Figure 9:**
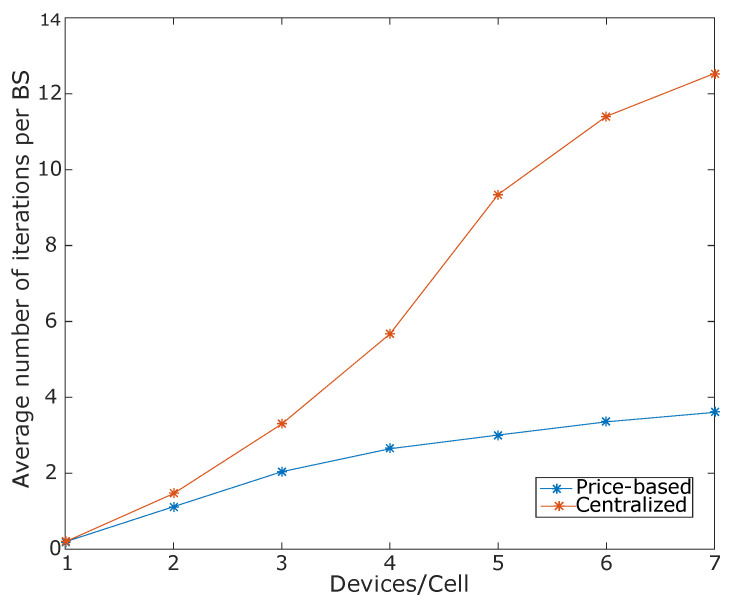
Average number of iterations required by each algorithm to solve the BS assignment problem.

Figure 9 presents an analysis of the number of iterations obtained by each algorithm under a variation of the number of devices per cell. The results have shown that our proposed solution requires fewer iterations to perform an update of the prices per resource unit. For instance, in the configuration of four devices per cell, our proposed approach requires an average of 2.5 iterations, while the centralized algorithm requires an average of 5.8 iterations. This means a reduction in the required number of iterations for about 56%, because of the centralized approach update at each iteration, the prices of only one BS. In contrast, in our solution, each BS independently updates its prices at each iteration.

## 6. Concluding Remarks

In this paper, we have studied the downlink BS assignment problem for scenarios that support HTC and MTC devices’ coexistence. Furthermore, we model an OFDMA-based cellular system limited by capacity on the radio and backhaul resources. This problem poses a combinatorial optimization problem amenable to be solved via traditional approaches as linear programming, though given the dynamism of the network, it is not always possible to ensure the compliance with all constraints. In this regard, we resorted to the dual version of the problem that allowed us to solved it without explicit parameterization in terms of the constraints making non-centralized and highly dynamic solution possible. At this point, the problem was reduced to that of finding an appropriate weighted utility $w_{ij}$ based on a distributed pricing approach. This results in a distributed pricing-based solution that runs at each BSs with scarce channel state information. The simulation results show that our proposed solution has a better performance than traditional Max-SINR and Min-PL, especially in terms of percentage of feasible solutions found and the system throughput. Additionally, the proposed pricing-based solution reduces the average cost at BSs due to the assignment solutions. Simultaneously, for the greedy approaches, there is observed a violation of the BSs capacity constraints. In future works, we plan to investigate how the edge computing resources could influence the decision-making process of the BS assignment problem in delay sensitive IoT applications with intensive computational requirements in mobile edge computing (MEC) scenarios.

## Figures and Tables

**Table 1:** Summary of base station (BS) assignment approaches in centralized (C) and distributed (D) control.

Ref.	Year	BS Assignment Approach			Devices	Multi-Cell	Resource Constraint (s)
		Control	Modelling	Core Mechanism			
[17]	2020	C	Heuristic	Iterative (radio load)	HTC	No	Radio
[18]	2020	C	Heuristic	Iterative (radio load)	HTC	No	Radio
[19]	2018	C	Heuristic	Iterative (radio load)	HTC	No	Radio
[20]	2017	C	Heuristic	Iterative (energy)	HTC	No	Radio, backhaul
[21]	2016	C	Heuristic	Kuhn-Munkres (energy)	HTC	No	Radio
[22]	2016	C	Heuristic	Iterative (radio load)	HTC	No	Radio
[23]	2016	C	Heuristic	Iterative (radio load)	HTC	No	Radio, backhaul
[24]	2014	D	Pricing	Price update (Coordinate descent)	HTC	No	Radio
[25]	2015	D	Pricing	Price update (Subgradient)	HTC	No	Radio
[26]	2013	D	Pricing	Price update (load-based)	HTC	No	Radio
[27]	2013	D	Pricing	Price update (load-based)	HTC	No	Radio
[28]	2015	D	Game theory	Cooperative (coalition strategies)	HTC, MTC	No	Radio
[29]	2016	D	Heuristic	Iterative (radio load)	HTC, MTC	No	Radio
[30]	2017	D	Price-based	Price update (Subgradient)	HTC, MTC	No	Radio
[31]	2017	D	Game theory	Cooperative (coalition strategies)	HTC, MTC	No	Radio
This work	2020	D	Price-based	Price update (Weighted utility)	HTC, MTC	Yes	Radio, backhaul

**Table 2:** Summary of Simulation Parameters.

Parameter	Value
Total number of cells *N*	19
Max. BS transmit power, $P_{j}^{\max}$	43 dBm
Cell radius	0.293 km
Operating frequency	2000 MHz
Reuse factor	3
Channel bandwidth	10 MHz
Number of data subchannels	1024
OFDM symbol duration	102.9 μs
Path loss model	$69.55+26.16\;\times \;\log_{10}\left(fc\right)-13.82\;\times \;\log_{10}\left(hb\right)-$ $ahm+\left(44.9-6.55\;\times \;\log_{10}\left(hb\right)\right)\;\times \;\log_{10}\left(d\right)$
BS height	30 m
Device height	1.5 m
Building penetration loss	10.6 dB
Shadowing standard deviation	8 dB
Thermal noise	−174 dBm/Hz
Receiver noise figure	5 dB
Backhaul capacity	4, 12, 21 Mbps
HTC devices rate requirements $R_{c}^{\min}$	100, 200, 250, 300 Kbps
MTC devices rate requirements $R_{d}^{\min}$	25 Kbps [36]
